# Trauma Ultrasound Training for Latin American Countries

**Published:** 2016-11-26

**Authors:** G Estebanez, AM Rubiano, AI Sánchez, J Ulloa, JC Puyana

**Affiliations:** Trauma and Emergency Service, Department of Neurosciences, Neiva University Hospital, South Colombia University, Colombia

**Keywords:** Competency, Education, Emergency, Latin América, Training, Trauma, Ultrasound

## Abstract

**Objective:**

The Pan-American Trauma Society (PTS) developed a Trauma and Emergency Ultrasound Course (USET) in response to the requirement for trauma ultrasound training for low-and middle-income countries. The objective of this study was to evaluate the efficiency of this course.

**Method:**

Pre- and post-course tests were used. And interval estimation of proportions was calculated at 95% CI. Theoretical and practical pre- and post-course knowledge were assessed with the Wilcoxon Signed Rank test at 0.05 level of statistical significance.

**Result:**

Between 2005 and 2007, 114 students, including general surgeons, emergency medicine physicians, anesthesiologists, critical care physicians, and residents of these specialties, were trained in seven countries (Uruguay, Peru, Mexico, Venezuela, Aruba, Colombia, and Ecuador). The difference on complete knowledge ranked scores before and after the course was statistically significant (p<0.001). After the course, almost all participants (97.4%) demonstrated complete knowledge in final evaluation.

**Conclusion:**

The USET course is an effective approach for trauma ultrasound training. Specific training programs for trauma care providers that work in low-and middle-income countries are necessary and could be performed with low cost training programs.

## Introduction

The successful management of trauma patients depends heavily upon the ability of the trauma doctor to perform rapid and accurate assessments to identify the need for, and expedite, potentially life-saving interventions. Physical examination alone, even when encompassing the monitoring and interpretation of patient’s vital signs, may not provide evidence of the presence of intra-abdominal bleeding necessitating emergency laparotomy [1].

The past 20 years has seen the utilization of FAST (Focused assessment sonography in trauma), in the immediate assessment of the trauma patient, become widespread [2]. This widespread acceptance, particularly in the United States, Asia, Australia and parts of Europe, was reflected by the American College of Surgeons’ (ACS) incorporation of FAST into the ATLS curriculum. The appeal of FAST is that it provides a rapid, accurate and easily repeated method of detecting, or “Ruling in”, the presence of free fluid within the pericardium or peritoneum [3]. Unlike diagnostic peritoneal lavage (PDL), FAST represents a non-invasive investigation that is without contraindication and yet has been shown to have similar accuracy in multiple studies [4,5].

In the developing world, or countries with low and middle income, FAST has the potential to play an even greater role in guiding trauma management as often CT scans may not be readily available [6]. The World Health Organization’s (WHO) guidelines for essential trauma care highlight the availability of ultrasound as “desirable”, however they also emphasize the importance of the presence of staff skilled in performing the procedure [7]. When considering that the accuracy, and thus the value, of FAST is largely operator dependent it is not surprising to discover that in numerous low income locations ultrasound machines are present but, due to lack of training, they are often not utilized [8].

Whilst organized and structured training programs in the use of ultrasound in the trauma setting are well founded in high-income countries there is a distinct lack in low and middle-income nations, particularly in Latin American countries (LAC). Whilst individuals in LAC may attend ATLS courses, these only discuss the theoretical application of ultrasound in the trauma scenario and thus only minimal, if any, practical experience of ultrasound in trauma may be acquired from attendance at these courses.

It was in response to this deficiency in training that the Pan-American Trauma Society (PTS) developed the Trauma and Emergency Ultrasound Course (USET). The aim of USET was to provide standardized and high quality ultrasound training to health care workers, of low-middle income countries, who may frequently be confronted with victims of trauma and medical emergencies.

The objective of this article is to evaluate the efficacy of the FAST module from the USET course in teaching course participants in seven countries (Uruguay, Peru, Mexico, Venezuela, Aruba, Colombia, and Ecuador) the theoretical and practical skills of ultrasound use in the trauma patient.

## Methods

The FAST module of the USET is a one-day course that was developed by members of the PTS including a trauma surgeon, a neurosurgeon, a vascular surgeon and an emergency physician. The USET course is structured as two separate modules. The first module consists of 8 hours of training (2 hours theory, 6 hours practical) in Focused Assessment by Sonography in Trauma (FAST); the second module (additional 8 hours) provides theoretical and practical teaching on the use of ultrasound in assisting the undertaking of various emergency room procedures including paracentesis, thoracic puncture, peripheral and central vascular access, novelty techniques for pneumothorax and intracranial pressure detection. Additionally a companion 187 pages USET handbook was developed for course participant reading. The chapters include: basic concepts of ultrasound (US), FAST, vascular US, venous thrombosis, arterial diagnose, abdominal aorta US, emergency procedures, general surgery US, emergency echocardiography, non-traumatic shock and US, pediatric US, soft tissue US, skeletal and muscle US, gallbladder US, gynecologic US, obstetric US, renal US, trans-cranial Doppler and future uses of US.

We analyzed the performance of 114 students to have undertaken the FAST module of the USET course, throughout seven LAC. Precourse assessments were undertaken to evaluate the existing level of knowledge and practical skills of the course participants. To assess the effectiveness of the course, with specific reference to this FAST module, post course assessments were undertaken. We evaluated student preand post-course performance with 10-question examinations.

Course participant’s pre and post-course tests results were analyzed using a Likert scale. A Likert scale for the knowledge degree was developed with choices from 1 to 5 (Table 1). Practical performance was evaluated by instructors using a Likert scale with possible choices of 1 to 2 during two practical sessions, one at the beginning of the course and the second one at the end.

Regarding assessment of participants’ practical skills, instructors completed appraisal forms which evaluated the ability of the course participant to successfully and independently obtain adequate views of all four FAST windows. A Likert scale ranging from 1 (uncomplete knowledge) to 2 (complete knowledge) was allocated to each participant according to whether all four FAST window views (PeriSpleen, Peri-Liver, Pelvic and pericardium) were effectively demonstrated. The course instructors determined scores in the skills stations by personal monitoring during the procedure.

We defined *effectiveness* of the course as the ability to improve student performance, through comparisons of post course and pre course assessments. Pre- and post-course knowledge tests’ results were summarized by reporting means, standard deviations (SD), medians, and inter-quartile ranges (IQR) and compared using non-parametric tests (Wilcoxon and Mann-Whitney). To provide a more useful and relevant interpretation, a description of all participants’ pre- and post-course tests’ results in the Knowledge tests and in the Practical skills tests were performed using absolute and relative frequencies for each of the Likert scale scores. Differences in proportions in Likert scale scores before and after the course were assessed with the Fisher exact test. An alpha threshold of 0.05 was set to determine statistical significance.

## Results

Between 2005 and 2007, 114 students, including general surgeons, emergency medicine physicians, anesthesiologists, critical care physicians, and residents of these specialties, were trained in seven countries (Uruguay, Peru, Mexico, Venezuela, Aruba, Colombia, and Ecuador). All participants were evaluated before and after the course in both knowledge and practical skills.

Before the course, four participants (3.5%) obtained the worst score and demonstrated lack of any knowledge during the initial evaluation (Likert scale score equal to 1), 18 participants (15.8%) scored incomplete partial knowledge (Likert scale score equal to 2), 53 participants (46.5%) scored partial knowledge (Likert scale score equal to 3), 35 participants (30.7%) scored almost complete knowledge (Likert scale score equal to 4), and four participants (3.5%) demonstrated a complete knowledge (Likert scale score equal to 5) (Table 2).

After the course, three participants (2.6%) demonstrated partial knowledge (Likert scale score equal to 3), 69 participants (60.5%) demonstrated an almost complete knowledge (Likert scale score equal to 4), and 42 participants (36.8%) demonstrated a complete knowledge (Likert scale score equal to 5) during the posttest. The differences in the proportions of participants scoring in the Likert scale scores between the pre and post-test were statistically significant (p-value<0.001).

Among all participants, the median Likert scale score in the knowledge evaluation before the course was 3 (inter quartile range [IQR], 3–4) and the median Likert scale score in the knowledge evaluation after the course was 4 (IQR, 4–5) (p-value<0.001) (Table 2).

Before the course, appropriate maneuvers during the practical skills evaluation were observed in 24 participants (21.5%). After the course, all 114 participants (100%) demonstrated appropriate maneuvers during the practical skills evaluation (p-value<0.001) (Table 2).

## Discussion

Much like the stethoscope, ultrasound is a genuinely useful, portable and rapidly useable diagnostic tool [9]. Emergency ultrasound can be utilized to diagnose acute life-threatening conditions, guide invasive procedures, treat emergency medical conditions and has ultimately improved the care of countless patients worldwide [10]. The widespread acceptance of the FAST exam in the trauma curriculum was confirmed with its inclusion within the ATLS course.

With improvements in the accessibility, speed and image quality of modern multi-detector CT scans, there is a growing body of opinion that CT should supersede FAST as the first line imaging of haemodynamically stable patients with blunt abdominal trauma (BAT) [11–13]. However, whilst FAST does not claim to rival the accuracy of CT in the detection of abdominal visceral injuries it does provide a rapid and accurate means of triaging patients based upon the detection of free abdominal fluid [2].

This is emphasized by several studies, which found that the use of FAST significantly decreased the time from arrival at the emergency department to arrival in the operating theatre [14–16]. Not only is ultrasound safe, rapid, and portable, it is also non-invasive, painless and unlike CT can be performed on haemodynamically unstable patients in the resuscitation area unlike CT [17]. Unlike formalized radiological investigations such as CT, the accuracy of ultrasound is heavily dependent upon operator competence and the adequacy of the training that they have received.

The USET course was established to provide formalized training in the use of ultrasound in trauma patients in low-middle income LAC areas that were previously without credentialed training on this topic. The FAST module of the USET training program is a one day course consisting of both didactic teaching and practical training in which positive findings of FAST are replicated using patients with peritoneal dialysis (PD).

It should be acknowledged that the use of PD patients in the teaching of FAST is not perfect, in that often the kidneys may be atrophied thus increasing the difficulty with which the typical FAST landmarks (Morrison’s and Keller’s pouches) are identified [18]. However, the benefits of using PD patients for the simulation of haemoperitoneum in FAST training are well appreciated and additionally its application has actually been shown to improve the learning curve of course participants [19].

The results of our study revealed that almost all (97.4%) of the 114 course participants demonstrated complete knowledge in the post-course theoretical and practical assessments. Through the assessment of participants post course practical skills, in addition to their theoretical knowledge, this enabled a comprehensive assessment of the efficacy of USET, compared to a similar smaller study in which only theoretical knowledge assessment was conducted [6]. Our results suggest that the USET course is effective in the education of both theoretical and practical aspects of ultrasound use in trauma.

The ideal structure and duration of effective FAST teaching is the subject of much debate. Recommendations regarding the duration of time required to teach participants in the use of FAST vary from 1 hour of practical experience to educational programs lasting 32 hours [20,21]. However, most studies support our findings that FAST can be taught effectively and efficiently during courses lasting only one day [22].

Whilst credentialed trauma ultrasound courses exist in high-income nations such as the USA, Canada and the United Kingdom, the participation costs are often in excess of $500. The costs of travel and course fees can be prohibitive to physicians and surgeons from Low and middle-income countries attending and attaining formalized credentialed ultrasound training. However, USET provides an affordable ($150) and locally undertaken quality standardized course through which they can effectively be taught the theoretical and practical aspects of ultrasound use in trauma.

Although we revealed that participants successfully demonstrated almost complete post course theoretical and practical trauma ultrasound knowledge, a limitation of this study is its lack of provision of any long term, post-course follow up. Obtaining objective data regarding the course participants’ utilization of FAST in their own clinical environment would be valuable to analyze the accuracy of FAST scans performed following course attendance.

The authors of this study are aware that whilst the USET course provides a comprehensive introduction of the use of ultrasound in trauma, upon completion of the course, participants are by no means experts in the application of this tool. Thus a significant amount of emphasis needs to be placed on ensuring that the participants continue to build on their acquired knowledge of FAST and hone their skills under quality supervision within their own clinical environments.

It is recommended that after completion of a FAST training course that participants must perform several observed examinations prior to being certified as competent in the application of this technique [23]. However, the required number of proctored exams is subject to much contention. One international consensus recommends that 200 supervised FAST examinations are required whereas other studies have concluded that satisfactory levels of accuracy are attained after performing only 10 supervised assessments [24,25]. This lack of clarity has highlighted the belief that certification of competence in the use of FAST should be based upon the individual’s competence in performing the skill and not on the number of times it has been performed [2].

Whilst we are unable to dictate the level of post course supervision that the participants of USET receive in their own clinical environment, we can conclude that upon completion of the course almost all were deemed as competent in both the theoretical and practical aspects of FAST. Reassuringly, it has shown that in developing countries, once the instructors have left, that participants of training courses continue to utilize their newly acquired ultrasound skills frequently and with a high level of accuracy [26].

## Conclusion

With the increasingly prominent role of ultrasound in the management of trauma patients there is an apparent need for standardized and cost effective teaching on FAST and the use of ultrasound in the trauma patient, particularly in countries with low and middle income [10,27].

We have demonstrated that the FAST module of the USET course is a cost effective and efficient means of teaching the use of FAST to large numbers of individuals, within low middle income LAC, and has the potential to improve the wide scale management of trauma patients.

## Figures and Tables

**Table 1:** Likert Scale description (Pre and post-test).

Likert Scale	Meaning
Don’t have any degree of knowledge	0 appropriate answers
Have an incomplete partial knowledge	1–3 appropriate answers
Have a partial knowledge	4–6 appropriate answers
Have a partial almost complete knowledge	7–9 appropriate answers
Have a complete knowledge	10 appropriate answers
Likert Scale Description (Practical station)	
Likert Scale	Meaning
Incomplete knowledge	Inappropriate maneuvers in the 4 windows view
Complete knowledge	Appropriate maneuvers in the 4 windows view

**Table 2:** Likert scale scores in knowledge test and practical skills test before and after the course.

Likert Scale	Pretest	Posttest	P-value
**Knowledge test**			
**Mean (SD)**	3.14 (0.85)	4.34 (0.52)	<0.001
**Median [IQR]**	3 [3–4]	4 [4–5]	
**Don’t have any degree of knowledge**	4 (3.5%)	0 (0.0%)	<0.001
**Incomplete partial knowledge**	18 (15.8%)	0 (0.0%)	
**Partial knowledge**	53 (46.5%)	3 (2.6%)	
**Partial almost complete knowledge**	35 (30.7%)	69 (60.5%)	
**Have a complete knowledge**	4 (3.5%)	42 (36.8%)	
**Practical skills test**			
**Incomplete knowledge**	90 (78.9%)	0 (0.0%)	<0.001
**Complete knowledge**	24 (21.1%)	114 (100%)	

SD: Standard Deviation; IQR: Inter Quartile Range
